# Cheating the Hunger Games; Mechanisms Controlling Clonal Diversity of CD8 Effector and Memory Populations

**DOI:** 10.3389/fimmu.2018.02831

**Published:** 2018-11-29

**Authors:** Inga Kavazović, Bojan Polić, Felix M. Wensveen

**Affiliations:** ^1^Department of Histology & Embryology University of Rijeka, Rijeka, Croatia; ^2^Department of Experimental Immunology, Amsterdam University Medical Center University of Amsterdam, Amsterdam, Netherlands

**Keywords:** immunity, CD8 T cell, affinity, memory, T cell receptor, differentiation, effector

## Abstract

Effector and memory CD8 T cells have an intrinsic difference in the way they must approach antigen; effector cells need to address the pathogen at hand and therefore favor outgrowth of only high-affinity clones. In contrast, the memory pool benefits from greater clonal diversity to recognize and eliminate pathogens with mutations in their immunogenic epitopes. Effector and memory fates are ultimately the result of the same three signals that control T cell activation; T cell receptor (TCR) engagement together with co-stimulation and cytokines. Great progress has been made in our understanding of the transcriptional programs that drive effector or memory differentiation. However, how these two different programs result from the same initial cues is still a matter of debate. An emerging image is that not only the classical three signals determine T cell differentiation, but also the ability of cells to access these signals relative to that of other activated clones. Inter-clonal competition is therefore not only a selective force, but also a mediator of CD8 T cell fate. How this is regulated on a transcriptional level, especially in the context of a selective “hunger game” based on antigen-affinity in which only cells of high-affinity are supposed to survive, is still poorly defined. In this review, we discuss recent literature that illustrates how antigen-affinity dependent inter-clonal competition shapes effector and memory populations in an environment of antigen affinity-driven selection. We argue that fine-tuning of TCR signal intensity presents an attractive target for regulating the scope of CD8 T cell vaccines.

## Introduction

CD8 T cells play a critical role in the protection of our body from the occurrence and recurrence of intracellular pathogens and tumors. To recognize the large number of potential threats, the naïve CD8 T cell pool consists of millions of clones, each unique based on its antigen receptor. To prevent an excessive use of resources for the maintenance of these cells, each clone is present at low frequency. Only upon activation do antigen-specific clones expand to form the effector and memory pools (1–4). Naïve CD8 T cells need three separate signals for optimal effector and memory generation: (1) antigen recognition by the T cell receptor (TCR), (2) co-stimulation, and (3) cytokines (5). These three signals are not hierarchically equal. Generally, only in case of TCR engagement do co-stimulation and cytokines contribute to T cell activation. Moreover, the affinity of the TCR determines the capacity of an activated cell to access vital co-stimulatory molecules, cytokines and nutrients (6). Considering the vast diversity of the naïve CD8 T cell pool, statistical probability dictates that for any given antigen, many more low- than high-affinity clones exist. To mount an efficient CD8 T cell response, selection of activated clones based on antigen-specificity must take place (6, 7). We have therefore proposed a fourth factor that controls effector and memory T cell formation: “competitive fitness”—the ability to compete for extracellular signals with other activated T cell clones based on antigen affinity (8).

The parameters that determine competitive fitness differ between effector and memory cells, because of the difference in the way that these pools must approach antigen. Upon infection, the effector pool is faced with an actively replicating pathogen and therefore only the most efficient, high-affinity clones are selected into its ranks (6, 9). Immunological memory must protect the host against re-infection with a previously encountered pathogen. Due to selective pressure on the original pathogen as it moves through its host population, re-infections are more likely to occur with a variant carrying mutations in its immunogenic epitopes (10–12). Hence, selection of memory clones is a trade-off between specificity and diversity. Too much specificity restricts antigen-recognition, which precludes responsiveness against mutated pathogens. Too much diversity impairs efficiency of recall responses. In both mice and humans increasing the diversity of the memory pool enables recognition of a larger fraction of the potential pathogen-carried sequence space resulting in a higher probability of recognizing mutated pathogens (13, 14). How clonal selection within the effector and memory cell populations is regulated is only partially understood. Here, we propose a crucial role of TCR signaling in an affinity-based inter-clonal competition which shapes clonal diversity and regulates effector and memory differentiation.

## The Impact of Signal Intensity on Cd8 T Cell Differentiation

The initiating event for CD8 T cell activation is recognition of an antigen embedded in the major histocompatibility complex (pMHC) on an antigen-presenting cell (APC) by the TCR. This results in the activation of a network of signaling cascades that mediate differentiation, proliferation, and survival (15, 16). Upon activation, a single naïve CD8 T cell has the potential to give rise to various effector and memory CD8 T cell subsets (17, 18). Divergent cell fates depend on the intensity of the cumulative signal activating an individual CD8 T cell (19). This signal strength represents the sum of different factors such as the affinity and avidity of TCR binding to antigen-pMHC complexes, co-stimulation, and cytokines (8, 20–22).

**Figure 1:**
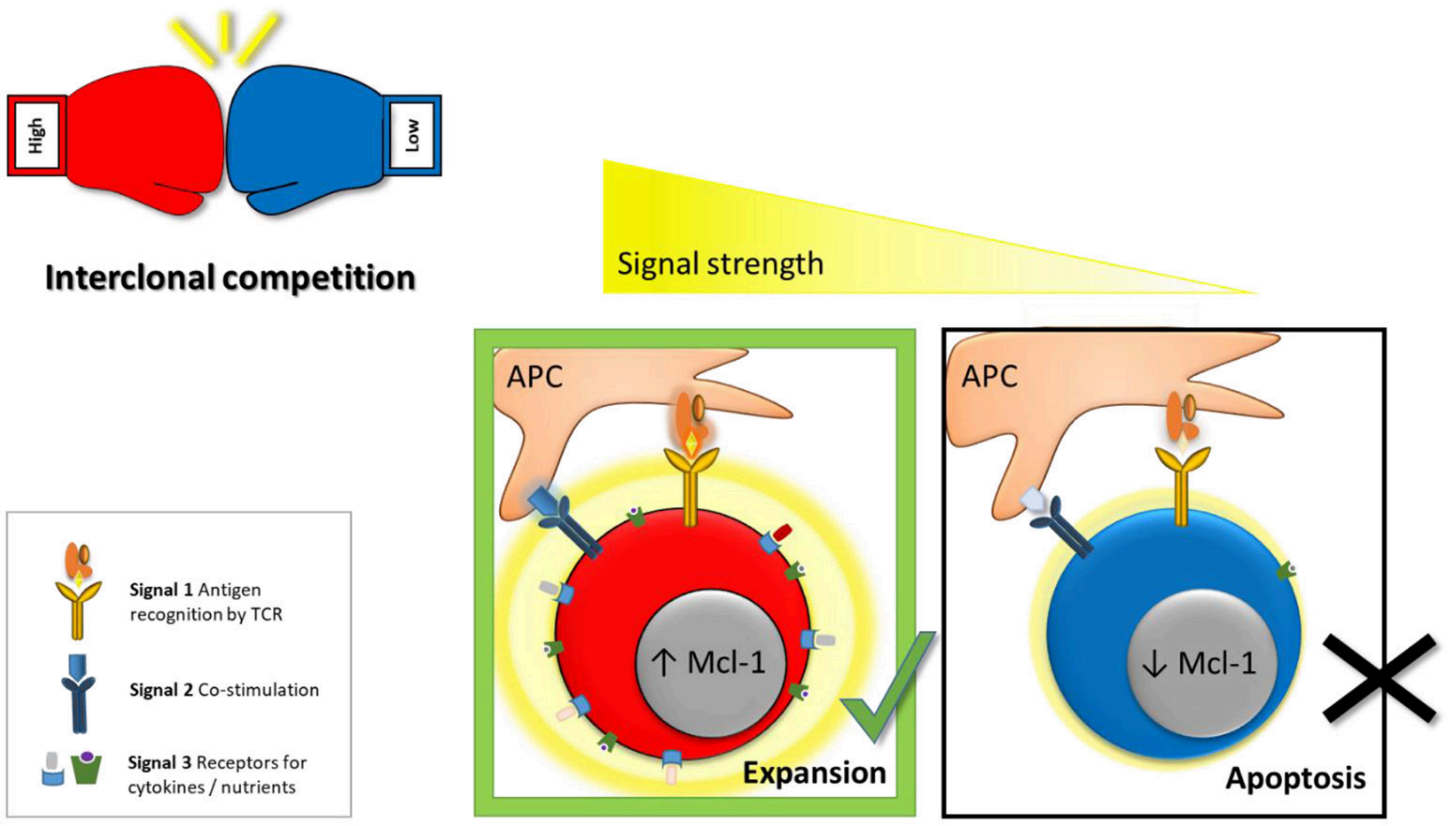
Model for inter-clonal competition between effector cells based on antigen-affinity. For efficient activation and optimal effector CD8 T cell formation 3 signals are required (1) antigen recognition by the TCR, (2) co-stimulation, and (3) cytokines. We proposed as a fourth factor “competitive fitness”—the ability to compete for these signals with other activated T cell clones. Cumulative signal strenght (visualized by a graded yellow halo) is the main factor controling the capacity of activated lymphocytes to access vital co-stimulatory molecules, cytokines and nutrients (e.g., glucose, amino acids). Thus, high-affinity effector cells have a competitive advantage over low-affinity cells in their ability to access these signals. In addition, high-affinity cells take-up more IL-2 which in turn mediates survival of high-affinity clones by triggering the PI3K signaling cascade and sustaining pro-survival proteins such as Mcl-1. Hence, low-affinity clones undergo negative selection through apoptosis to ensure that only the fittest, high-affinity clones contribute to the antiviral response.

Initially, it was proposed that only a cumulative signal of high overall strength allows T cell activation and formation of effector and memory cells (23). This was based on the observation that only cells of high-affinity vigorously expand upon activation (24). The model was challenged by the finding that even very weak TCR-pMHC interactions promote proliferation and generation of functional memory (25, 26). In addition, even a brief 2 h priming phase was shown to be sufficient to induce the complete diversity of effector and memory CD8 T cell subsets (27–29). To analyze these processes more directly, SIINFEKL (Ova)-specific OT-1 cells were transferred to naïve recipient mice, which were subsequently infected with *L. monocytogenes* (LM) expressing Ova or altered peptide ligands (APL) that bind the OT-1 TCR with lower affinity. This revealed that even weak ligands are sufficient to activate naïve cells and mediate formation of both effector and memory T cells (30). This raised the question how the immune system prevents that clones of low specificity and efficiency expand and exhaust the limited amount of available resources. The answer came from the observation that the potency to induce effector cell proliferation positively correlates with the intensity of the TCR signal (24, 30–32). Decreasing the cumulative signal strength by pretreating mice with antibiotics before *L. monocytogenes* infection and thus lowering antigenic load resulted in reduced expansion of antigen specific effector T cells (33, 34). In addition to a proliferative advantage of high-affinity cells, activated effector CD8 T cells were shown to undergo negative selection of low-affinity clones based on a reduced capacity of these cells to access and thus outcompete other clones for limited resources (8). Upon activation T cells induce expression of the IL-2 receptor in an antigen-affinity dependent manner (6, 30). IL-2 mediates survival by triggering the PI3K signaling cascade and sustaining the pro-survival protein Mcl-1 (Figure 1). High-affinity effector cells therefore have a competitive survival advantage over low-affinity cells in their ability to access IL-2. This selection process narrows clonal diversity, since only highly specific clones are allowed to generate progeny and create an almost monoclonal effector CD8 T cell pool (6, 8). Animals lacking Noxa, a pro-apoptotic antagonist of Mcl-1, have a reduced survival threshold for effector cells and therefore showed reduced dependency on IL-2. As a result, these mice had an increased number of low-affinity clones contributing to the effector pool, which was of reduced anti-viral potential (6).

Co-stimulation and cytokines greatly contribute to the cumulative activating signal intensity and therefore have a major impact on TCR-affinity mediated selection of CD8 T cell clones. CD28-driven co-stimulation is essential for proper CD8 T cell responses after weak TCR-pMHC interactions. Conversely, high antigen doses and prolonged antigen stimulation can compensate for a lack of CD28 co-stimulation *in vivo* (35, 36). CD27-driven co-stimulation promotes production of IL-2 in activated T cells (37). Animals deficient for CD27 therefore have reduced access to IL-2, resulting in a less clonally diverse effector response of increased overall affinity (13). Notably, expression of CD70, the ligand of CD27, is regulated by antigen avidity (13, 38–40), but whether this contributes to the diversity of the effector response is unknown. Similarly, cytokines impact cell fate decisions and clonal selection mechanisms. CD8 T cells activated in the presence of high levels of IL-2 or IL-12 exhibit increased proliferation rates and superior effector functions (23, 30, 33, 41–43). Exogenous addition of IL-2 rescued survival of low-affinity cells (6), indicating that stronger inflammatory responses will allow for more clones to contribute to the effector response, though this does not necessarily promote their dominance.

In summary, TCR signaling is not an on/off switch. Rather, it enables integration of signals with different intensities, which are further amplified by the right cytokines and co-stimulatory molecules. Fine-tuning of TCR signal intensity shapes T cell differentiation and clonal selection.

## Inter-Clonal Competition in the Context of Effector and Memory Formation

Even though a naïve cell can generate both effector and memory cells (44), memory potential is associated with weaker activating signals. Very low affinity antigens are still able to induce memory formation but have a strongly reduced capacity to induce effector differentiation (30, 45). Exogenous factors such as IL-2, IL-12, or CD28 co-stimulation add to the cumulative activating signal and help activated cells to obtain an effector phenotype (43, 46). Very high levels of stimulation, in contrast, push T cells “beyond” an effector stage into exhaustion (47, 48). Various models have been proposed how activating signal strength regulates CD8 memory formation. The “decreasing potential” model suggests that memory formation is the “default” state of activated T cells and that effector memory or effector cell differentiation is only possible if a certain level of activation is reached(49, 50). Whether this level represents a binary threshold, or whether effector potential is gradually increased in response to increasing signal strength is a matter debate and appears to depend on the molecules that are used to determine threshold values(43, 51–55).

Mostly, the impact of affinity on effector and memory potential has been interrogated by presenting a single (TCR-transgenic) T cell clone, with high- or low-affinity ligands (30, 45). However, a biologically more relevant question is how signal strength is linked to memory formation, not at the level of a single clone but in the context of the entire antigen-responsive population. Statistical probability dictates that for a given antigen, many more high-affinity than low-affinity cells exist within the naïve T cell pool. Hence, molecular mechanisms are in place to ensure that preferentially cells of high-efficiency are selected into both the effector and memory cell pools (6–8, 13). The impact of cumulative signal intensity is therefore not only a checkpoint controlling effector vs. memory fate decisions, but also controls the competitive fitness of cells in a selective environment that regulates the diversity of antigen-experienced T cell populations. To shed more light on this concept, experiments were performed in which a pool of individually labeled OT-1 cells was transferred to a host which was subsequently infected with LM-Ova. Analysis of donor cells revealed that even within a monoclonal high-affinity population, a relatively small fraction of clones dominates the effector response (17, 18). This would suggest that only a small number of cells reaches the cumulative signaling threshold required for CD8 T cell expansion. When a sufficiently high number of monoclonal cells is transferred, stochastic effects are negated, which ensures that in experimental settings donor cells usually make a significant contribution to the effector response (18). However, in a physiological setting, each clone is present at very low frequency (3). This indicates that inter-clonal competition becomes an important factor that controls shaping of the antigen-specific cell pool. Indeed, when mice were transferred with only a single OT-1 cell, in less than one third of animals these cells could be recovered after infection with LM-Ova (18). The recruitment of antigen-specific cells into the immune response is highly efficient and nearly complete (56), excluding limited antigen-exposure as a determining factor. Thus, considering the fact that effector cells are derived from a small number of precursors that is able to generate exponential expansion (17, 18), small differences in competitive fitness will ensure highly selective outgrowth of clones.

**Figure 2:**
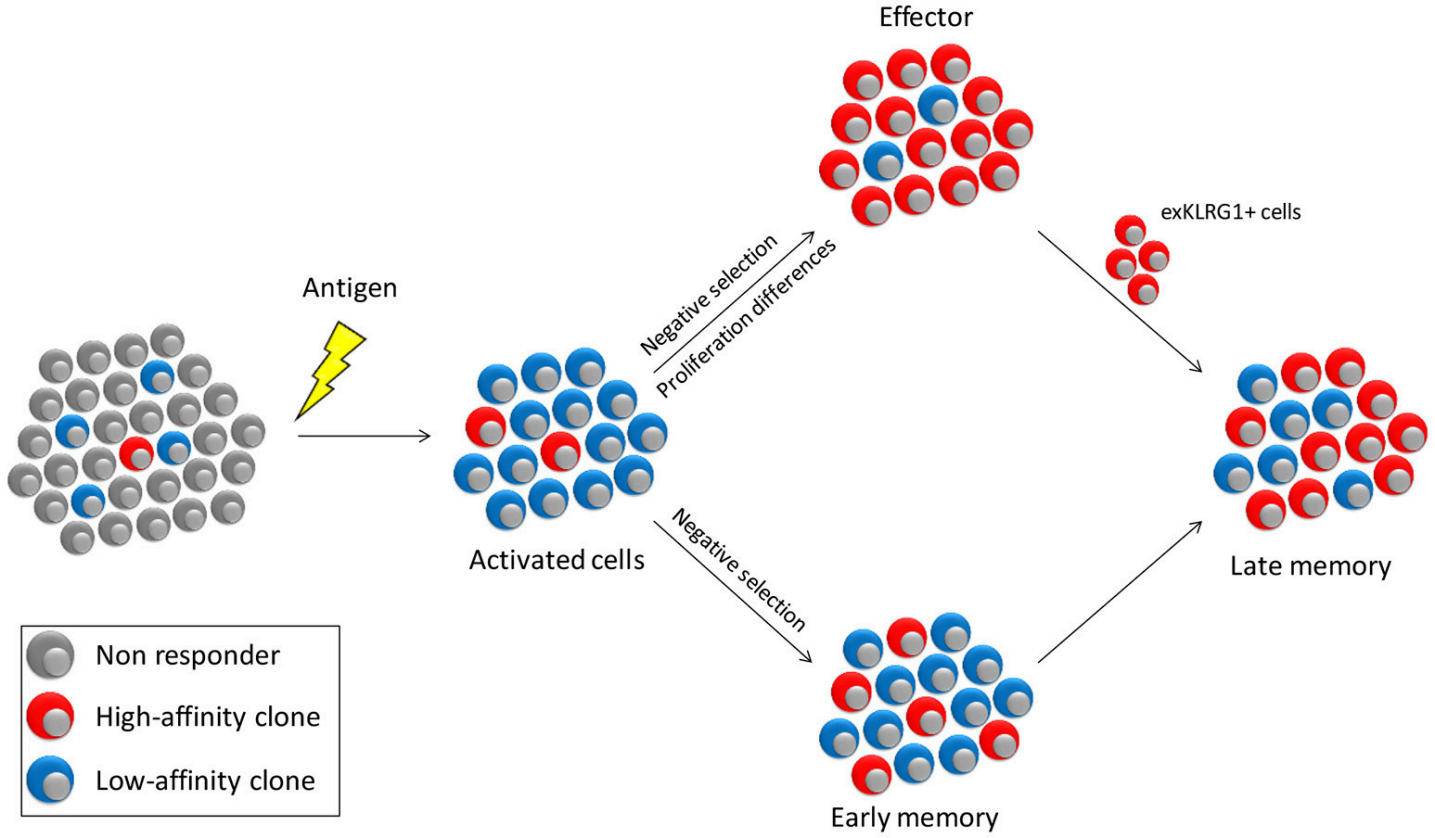
Model for affinity selection within the effector and memory pools. Antigen encounter will activate a small number of high affinity cells and a much larger number of cells with lower affinity. The effector pool is stringently selected for cells of the highest affinity both through negative selection of low-affinity cells and through a proliferative advantage of high- over low-affinity cells. In contrast, affinity-based selection of early memory is less strong, since differences in proliferation between high- and low-affinity cells is less pronounced. Selection of high-affinity cells will therefore primarily occur due to competition for survival factors such as CD27 co-stimulation and cytokines. Late memory is supplemented by exKLRG1 cells, which are predominantly of high-affinity, thus increasing the overall affinity of the memory pool.

In the cell-tracing experiment, cells that did not undergo massive expansion generally adopted a memory-like phenotype (18). Together with the observation that low-affinity cells preferentially form memory, the question arises whether only the effector pool is selected for high-affinity clones and that the memory pool allows contribution of all activated cells. Studies in which the clonal diversity of effector and memory cells was directly compared showed that the effector cell pool is much more restricted in its clonal diversity than the memory pool directed against the same antigen (7, 13, 57). However, clones that dominate the effector pool are also dominant in the memory population, albeit to a lower degree (7, 13, 57). Low cumulative signal strength favors memory formation and is associated with reduced proliferation (30, 31). Why then, is the memory response not completely dominated by low-affinity cells? One possibility is that high-affinity cells have a selective advantage also during memory formation. Another option is that they preferentially use a different mechanism to form memory than low-affinity cells. These models are not mutually exclusive and experimental evidence for both exist (Figure 2). Mice deficient for the co-stimulatory molecule CD27 generate a memory pool of comparable size as wild type controls yet is almost devoid of low-affinity clones (13). Similarly, low-affinity cells have a higher dependence on TNF receptor signaling during recall (58). This indicates that low-affinity memory precursors have increased dependence on factors that contribute to the cumulative activating signal and thus have a survival disadvantage when competing for these factors.

Antigen-experienced cells can be subdivided based on different parameters, but a common segregation uses IL-7Rα (CD127) and KLRG1. Memory precursors (MPECs) are defined as CD127^+^KLRG1^−^ whereas short lived effectors (SLECs) have the converse phenotype. A recent study indicates, however, that with the CD127^+^KLRG1^+^ cell pool and even among SLECs, cells exist that form “exKLRG1^+^” memory after clearance of a pathogen (59). Even though the frequency of cells with memory potential in these pools is much lower than amongst MPECs, the high number of KLRG1^+^ cells formed during an immune response ensures that in absolute numbers exKLRG1^+^ cells make a significant contribution to the memory pool (59). High-affinity cells preferentially form cells with a SLEC phenotype, whereas low-affinity cells more rapidly become MPECs (30, 45). Even though direct experimental evidence is still lacking, these findings indicate that low-affinity memory cells are formed directly, whereas high-affinity memory is also derived from exKLRG1^+^ effector type cells (Figure 2).

Maintenance of CD8 memory cells is independent of antigen and predominantly depends on cytokines such as IL-15 and IL-7 (60, 61). Whereas expression of cytokine receptors differs between cells of high- and low-affinity early after activation, at later time-points these differences are lost (6, 30). In the first weeks after clearance of a pathogen, the avidity of the antigen-specific pool therefore still changes as long-lived effector cells undergo apoptosis (62). However, once the clonal composition of the memory pool is established it remains stable for months to years after initial infection, both in humans and mice (63–66). Thus, clonal diversity of the memory CD8 T cell pool appears to be a long-term investment of the immune system to counter viral mutants.

An open question is how memory cell formation is influenced by inter-clonal competition on a molecular level. Various factors important for effector cell formation are induced in a way that directly correlates with antigen affinity, such as T-bet, IRF4, and Blimp-1 and these suppress expression of memory-associated molecules, such as Eomes and Tcf7 (54, 67, 68). The transcription factor IRF4 was found to regulate expansion of effector cells by promoting the metabolic switch to aerobic glycolysis in a TCR affinity–dependent manner. IRF4 expression was higher in high-affinity clones, ensuring their preferential expansion and effector differentiation over low-affinity clones (69, 70). Surprisingly, both Eomes and Tcf7 are induced upon activation of T cells (71, 72) and expression of Eomes can even be higher in high- than in low-affinity cells, dependent on the level of stimulation (45, 70). Notably, both T-bet and Eomes are essential for CD8 T cells to obtain a normal effector cell phenotype (72). The ratio between these molecules, rather than their expression level therefore appears to determine whether a cell obtains a memory or effector cell phenotype (46). How this dynamic regulation of transcription factors is regulated in the context of affinity-based selection in effector and memory cell pools remains to be elucidated.

In summary, the impact of cumulative signal intensity on effector vs. memory cell differentiation should be viewed in the context of clonal selection strategies that shape the antigen-specific cell pools. The impact of affinity on cell fate decisions appears to have evolved in order to ensure selection of only highly specific cells in the effector cell pool, whilst allowing sufficient diversity of CD8 T cell memory in a pool that is still dominated by high-affinity cells.

## Therapeutic Potential of Clonal Diversity

Enhanced diversity within the memory CD8 T cell pool is of particular benefit against re-infections with rapidly mutating viruses (73). For example, HIV patients appear to benefit from greater clonal diversity of their virus-specific T cell response (13, 14, 73–77). As an effective vaccine against HIV remains elusive, future strategies may involve manipulation of IL-2 levels and/or co-stimulatory molecules during priming to broaden the scope of the immune response. Evolutionary, increased clonal diversity of the memory pool compared to the effector pool is an acceptable strategy, as it does not appear to greatly reduce recall capacity against the original antigen. *Cd27*^−/−^ mice, which generate a memory CD8 T cell pool that almost exclusively consists of high-affinity cells do not show an increased recall response following re-infection with a pathogen carrying high-affinity ligands (13). Similarly, co-transfer of high- and low-affinity memory cells directed against the same antigen does not result in a reduced ability of high-affinity cells to expand upon antigen re-encounter (45). In fact, re-encounter of the same antigen further skews the secondary effector pool in favor of high-affinity clones. In addition, re-infection with pathogens carrying a mutated immuno-dominant epitope promotes selective outgrowth of previously low-frequency clones that have now become of high-affinity (13, 65). Thus, clonal selection plays an important role both during primary and secondary responses, but does not affect functionality of subdominant clones. Increasing memory diversity of a vaccine against pathogens is therefore unlikely to reduce the overall effectiveness of protection.

Vaccination against tumors should target only transformed cells while avoiding unnecessary damage of healthy tissue. Reducing the number of targeted epitopes included in a vaccine lowers chances of off-target effects, but also limits the effectiveness of a vaccine and allows for more rapid outgrowth of cells with mutations in their immunogenic epitopes. Rather, anti-tumor vaccination in combination with a strategy that narrows the scope of the immune response per epitope holds promise for a more efficient and specific treatment. A better understanding of the molecular mechanisms that control the diversity of the T cell response are therefore of crucial importance (8, 78, 79).

The degree of heterogeneity within the CD8 T cell response depends on the ability of activated clones to integrate signals from the TCR, co-stimulatory molecules and cytokines, but also their relative fitness in an environment of rapidly expanding cells competing for the same resources. Recent studies demonstrate the importance TCR signal strength in regulating T cell differentiation, but much remains unknown about the molecular mechanisms that control the clonal selection strategies that shape the diversity of the effector and memory pools. Deeper insight in the transcriptional network underlying affinity-based clonal selection therefore holds great promise for the development of novel, more efficient CD8 T cell vaccines with an altered scope.

## Author Contributions

IK and FMW wrote the manuscript. IK, FMW, and BP participated in drafting and editing the text and figures. All authors gave final approval to the version submitted.

### Conflict of Interest Statement

The authors declare that the research was conducted in the absence of any commercial or financial relationships that could be construed as a potential conflict of interest.
